# Quality of care for community-dwelling older adults living with dementia in BC: an interrupted time-series analysis to examine the effect of the COVID-19 pandemic

**DOI:** 10.23889/ijpds.v9i5.2719

**Published:** 2024-09-18

**Authors:** Mary Helmer-Smith, Kimberlyn McGrail, Sabrina Wong, Michael Law

**Affiliations:** 1 University of British Columbia

## Objective

Quality care for older adults living with dementia (OALwD) is a priority in Canada, nationally, and British Columbia (BC), provincially. The COVID-19 pandemic likely changed care quality, but this has not been assessed. We are examining the effect of pandemic-related policy changes on quality of care for community-dwelling OALwD in BC.

## Approach

This population-based retrospective cohort study uses linked administrative data to examine trends in population-level indicators of quality care from 2016-2023. We will compare effects among community-dwelling BC residents aged 65+ with dementia and those without dementia. Indicators including hospitalization rate and medication use were selected, based on the Institute of Medicine’s six dimensions of quality (e.g., effectiveness, safety). Interrupted time series (ITS) analysis will examine changes between “pre-pandemic” and “in-pandemic” periods. Equity will be assessed through stratification by sociodemographic variables.

## Anticipated Results

Percent change in the level and slope of each indicator’s trend will be reported. Single ITS will identify the effect of the pandemic on each outcome in the dementia group only. Controlled ITS will identify whether changes differ from those observed in older adults without dementia. Together, observed changes in the indicators and differences across equity factors will inform an interpretation of the overall effect of the pandemic on quality of care for community-dwelling OALwD in BC.

## Implications

We will determine whether quality of care changed for older adults because of the pandemic and whether changes differed for OALwD. This knowledge will inform practice and policy for delivery of high quality, proactive dementia care.

